# Peroxiredoxin-6 regulates p38-mediated epithelial–mesenchymal transition in HCT116 colon cancer cells

**DOI:** 10.1186/s40709-021-00153-6

**Published:** 2021-11-23

**Authors:** Unbin Chae, Bokyung Kim, HanSeop Kim, Young-Ho Park, Seung Hwan Lee, Sun-Uk Kim, Dong-Seok Lee

**Affiliations:** 1 Futuristic Animal Resource & Research Center, Korea Research Institute of Bioscience and Biotechnology (KRIBB), Cheongju, 28116 Republic of Korea; 2 School of Life Sciences, BK21 FOUR KNU Creative BioResearch Group, Kyungpook National University, Daegu, 41566 Republic of Korea; 3 Department of Functional Genomics, KRIBB School of Bioscience, Korea University of Science and Technology (UST), Daejeon, 34113 Republic of Korea; 4 National Primate Research Center (NPRC), Korea Research Institute of Bioscience and Biotechnology (KRIBB), Cheongju, 28116 Republic of Korea

**Keywords:** Colon cancer, Epithelial–mesenchymal transition (EMT), HCT116, p38, Peroxiredoxin 6 (Prx6)

## Abstract

**Background:**

Peroxiredoxins (Prxs) are antioxidant enzymes that protect cells from oxidative stress induced by several factors. They regulate several signaling pathways, such as metabolism, immune response, and intracellular reactive oxygen species (ROS) homeostasis. Epithelial–mesenchymal transition (EMT) is a transforming process that induces the loss of epithelial features of cancer cells and the gain of the mesenchymal phenotype. The EMT promotes metastasis and cancer cell progression mediated by several pathways, such as mitogen-activated protein kinases (MAPKs) and epigenetic regulators.

**Methods:**

We used Prx6 overexpressed and downregulated HCT116 cells to study the mechanism between Prx6 and colon cancer. The expression of Prx6, GAPDH, Snail, Twist1, E-cadherin, Vimentin, N-cadherin, ERK, p-ERK, p38, p-p38, JNK, and p-JNK were detected by Western blotting. Additionally, an animal study for xenograft assay was conducted to explore the function of Prx6 on tumorigenesis. Cell proliferation and migration were determined by IncuCyte Cell Proliferation and colony formation assays.

**Results:**

We confirmed that the expression of Prx6 and EMT signaling highly occurs in HCT116 compared with that in other colon cancer cell lines. Prx6 regulates the EMT signaling pathway by modulating EMT-related transcriptional repressors and mesenchymal genes in HCT116 colon cancer cells. Under the Prx6-overexpressed condition, HCT116 cells proliferation increased significantly. Moreover, the HCT116 cells proliferation decreased in the siPrx6-treated cells. Eleven days after HCT116 cell injection, Prx6 was overexpressed in the HCT116-injected mice, and the tumor volume increased significantly compared with that of the control mice. Furthermore, Prx6 regulates EMT signaling through p38 phosphorylation in colon cancer cells.

**Conclusion:**

We suggested that Prx6 regulates EMT signaling pathway through p38 phosphorylation modulation in HCT116 colon cancer cells.

**Supplementary Information:**

The online version contains supplementary material available at 10.1186/s40709-021-00153-6.

## Background

Reactive oxygen species (ROS), including hydrogen peroxide (H_2_O_2_), are highly reactive and can cause oxidative stress during cellular metabolism [1]. Therefore, ROS regulation is essential for healthy cellular metabolism, for which, most cells possess superoxide dismutase, catalase, and peroxiredoxin. The redox balance is known for maintaining the balance between ROS and antioxidant enzymes [2]. It is supported by the capacity of the antioxidant defense system in cellular metabolism [3]. At an optimum level, ROS, underregulated by antioxidant enzymes, functions as a mediator of cell growth [4], metabolism [5], signal messenger [6], and maintaining redox balance [7]. If ROS overwhelms the capacity of the cellular antioxidant defense system, excessive oxidative stress would destroy DNA, lipids, and proteins [8]. It promotes abnormal cell growth and proliferation, in case ROS level is out of control due to antioxidants. Compared with normal cells, oxidative stress is highly increased in cancer cells because of excessive ROS generation [9]. Furthermore, this enhanced oxidative stress can lead to tumor cell growth and metastasis [10]. Therefore, the regulation of antioxidants is closely related to cancer cell growth and proliferation.

Peroxiredoxins (Prxs) are powerful antioxidant enzymes that remove hydroperoxide, known as intracellular ROS [11]. The Prxs have six isoforms and are divided into three types by their structure and reduction processes, i.e., typical-2-Cys, atypical-2-Cys and atypical-1-Cys. Prx1, 2, 3, and 4 are typical-2-Cys Prx, and Prx5 is an atypical-2-Cys Prx. In contrast, 2-Cys Prx uses thioredoxin for its reduction, whereas Prx6, a 1-Cys Prx, is the only Prx that uses glutathione for the same process [12]. Like other Prxs series, many studies regarding Prx6 and diseases induced by oxidative stress have already been done. Moreover, studies have reported that Prx6 makes cancer cells sensitive in chemotherapy, resulting in apoptosis [13]. However, compared with other cancers, there are not many studies that have evaluated the roles of Prx6 in colon cancer development. Therefore, it is necessary to study the function and mechanism of Prx6 in the proliferation and growth of colorectal cancer.

The epithelial–mesenchymal transition (EMT) is described as a process in which the less motility epithelial cells are transformed into mesenchymal cells that gain motility [14]. The EMT can be found in embryonic development [15], fibrosis of tissues [16], and metastasis of cancer [17]. Studies have demonstrated that EMT is associated with cancer progression and metastasis. For the EMT to occur in cancer cells, genes, such as Snai1, Twist1, and Zeb1, need to be expressed [18]. Furthermore, epithelial marker proteins, such as E-cadherin, occludin, and type-4 collagen are decreased in cancer cells when EMT occurs [19]. These factors result in more invasive and metastatic cancer cells, thereby making cancer therapies difficult. In contrast, gene transcription factors of EMT (Snai1, Twist1, and Zeb1) affect the epithelial features of the cells. Also, they induce an increase in mesenchymal features. Therefore, the regulation of EMT signaling through modulation of transcription factors is a critical alleviation factor of cancer progression and metastasis.

The mitogen-activated protein kinases (MAPKs) are specific for serine and threonine involved in various cellular responses, i.e., proliferation, gene expression, and cell survival. In mammalian cells, MAPKs are primarily defined into three subgroups: the extracellular signal-regulated kinases (ERKs, including ERK-1 and ERK-2), the c-Jun N-terminal kinases (JNKs, including JNK-1, JNK-2, and JNK-3), and the p38 MAPKs (including p38-α, p38-β, p38-γ, and p38-δ). The MAPK activation is stimulated by proinflammatory cytokines [20], heat shock [21], and oxidative stress [22]. As one of the well-known MAPK stimulators, ROS activates them, serving as the cellular second messenger that enables the well-controlled proliferation and growth of cells [6]. However, excessive ROS inhibits cell growth and proliferation with MAPKs, leading to apoptosis [23]. Furthermore, among the MAPKs pathway, the p38 pathway plays an essential role in inhibiting EMT [24]. By maintaining E-cadherin through tak1-NF-κb signaling, p38 inhibits EMT. Therefore, the p38 pathway regulation through the ROS-level modulation is a fascinating research avenue for cancer therapy studies.

Therefore, Prx6 is a powerful antioxidant enzyme used for cancer therapy through ROS regulation. However, in the case of colon cancer, the capacity of Prx6 to regulate EMT signaling in colon cancer cell is still unknown. Therefore, in this study, we investigated the relationship between ROS regulation by Prx6 and EMT in colon cancer cells.

## Methods

### Materials

The U0126 inhibitor was obtained from Santa Cruz Biotechnology Inc. (Dallas, TX, USA). The SB203580 and SP600125 inhibitors were obtained from Cell Signaling Technology (Danvers, MA, USA).

### Cell lines and cultures

Human colorectal cancer cell lines—HCT116, HT15, HT29, and SW480—and human colon cancer cells were purchased from the American Type Culture Collection (Manassas, VA, USA). As per the supplier protocol, the cell lines were maintained in RPMI-1640 supplemented with 10% (v/v) fetal bovine serum (FBS). HCT116 and SW480 cells were grown to 60% confluency and then transfected with 10 pmol of siRNA against Prx6 (siPrx6; Bioneer, Daejeon, Korea).

### Transfection and selection of cells with stable expression

For all cell lines, 1 × 10^5^ cells were seeded onto six-well plates. Then, after 24 h, cells were transfected with 2-μg pLenti6.3-Prx6 using Effectene (Qiagen, CA, USA) according to the manufacturer instructions. After another 24 h, the transfected cells were selected by supplementing the culture medium with 8 mg ml^−1^ blasticidin (Invitrogen, MA, USA).

### Western blotting

Protein lysates were prepared using an ice-cold PRO-PREP protein extraction solution (iNtRON Biotechnology Inc., Seongnam, Korea). For all samples, 20 μg protein lysates was separated on SDS–polyacrylamide gels, transferred onto nitrocellulose membranes (Pall Corporation, NY, USA), and blocked with 5% skim milk. The samples were analyzed, as per the manufacturer instructions, using the following primary antibodies: Twist1 (Cell Signaling Biotechnology), PRX6 and Vimentin (AbFrontier, Seoul, Korea), E-cadherin (BD Biosciences, NJ, USA), and Snail and GAPDH (Santa Cruz Biotechnology Inc.). After washing off the excess secondary antibodies (horseradish peroxidase-conjugated goat antirabbit, mouse, and donkey antigoat, obtained from Thermo Scientific, IL, USA), specific banding was detected using Clarity Western ECL Substrate (Bio-Rad, CA, USA).

### Xenograft assay

Five-week-old female nude mice (BALB/c-nu) were purchased from Central Lab Animal Inc. (Seoul, Korea). All animal experiments were approved and conducted following the guidelines of the Animal Care Committee of Kyungpook National University. Experiments were conducted in the animal research laboratory in the College of Natural Sciences, Kyungpook National University. When the mice were six weeks old, 5 × 10^6^ cells resuspended in 100 μL phosphate-buffered saline (PBS) were subcutaneously injected using a 31-gage needle (n = 3). The sizes of the resulting tumors were measured using a caliper and calculated using the following formula: W (width)^2^ × L (length)/2. Then, the mice were sacrificed under deep anesthesia using isoflurane after measuring the tumor mass from day 21.

### Foci formation

Cell lines were seeded on 6-well plates. Ten days after seeding, the cells were washed twice with PBS, fixed with 4% paraformaldehyde (Sigma, Saint Louis, MO, USA), and stained with 2% crystal violet (Sigma, Saint Louis, MO, USA).

### Proliferation assay

Cells were seeded onto 96-well ImageLock plates (Essen Bioscience, Ann Arbor, MI, USA) at a density of 2 × 10^4^ cells per well and incubated in RPMI-1640 with 10% standard FBS for 56 h to measure cell proliferation. Furthermore, the plates were scanned on the IncuCyte imager (Essen Bioscience, Ann Arbor, MI, USA), and the data were analyzed using the IncuCyte Cell Proliferation assay software. Results are representative of three independent experiments.

### Statistical analysis

Data were shown as the mean and standard deviation (SD) of three independent experiments (n = 3). Differences observed between experimental groups were tested for statistical significance using one-way and two-way ANOVA followed by Tukey HSD post-hoc test with GraphPad Prism (GraphPad, San Diego, CA, USA). *P* < 0.05 was considered statistically significant and is shown in the figures by an asterisk, while *p* < 0.01 and *p* < 0.001 are shown by two and three asterisks, respectively.

## Results

### Peroxiredoxin 6 and EMT-related genes are highly expressed in HCT116 cells

**Fig. 1:**
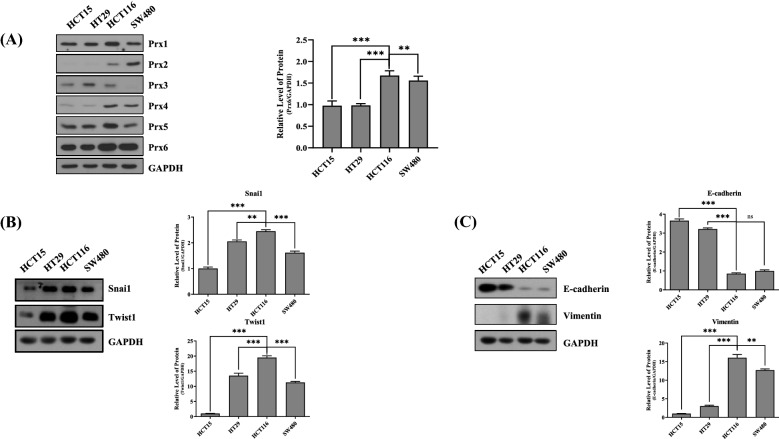
Expression levels of Prx6 and EMT-related genes in colon cancer cells. **A** Western blotting of Prx6 in various cancer cell lines, such as HCT15, HT29, HCT116, and SW480. **B** Western blotting of EMT-transcription factors (Snail, Twist1) in cancer cell lines. **C** Western blotting of EMT-marker proteins, E-cadherin and Vimentin, in cancer cell lines. Graphs represent the quantification of Western blot band intensity. Data are expressed as mean ± SD (n = 3). *p < 0.05, **p < 0.01, and ***p < 0.001

First, Prx series in four colon cancer cell lines were screened for detecting Prxs effect on colorectal cancer cell growth (Fig. 1A). Among the six Prx series, Prx6 exhibited a slightly high expression than other Prxs. Furthermore, HCT116 and SW480 cell lines had higher Prx6 protein expression levels than other colon cancer cells. Next, we confirmed the expression levels of Snail and Twist1 genes, which are well-known EMT-transcription factors. The EMT-transcription factor expression levels were the highest in the HCT116 compared with those in other colon cancer cells (Fig. 1B). Furthermore, E-cadherin and Vimentin, which are well-known EMT-marker proteins, were detected by Western blotting. As expected, the lowest E-cadherin and the highest Vimentin expression levels were indicated in HCT116 (Fig. 1C). Thus, we confirmed that the expression of Prx6 and EMT signaling occurs highly in HCT116 compared with that in other colon cancer cell lines.

### Peroxiredoxin 6 regulates EMT-related genes expression in HCT116

**Fig. 2:**
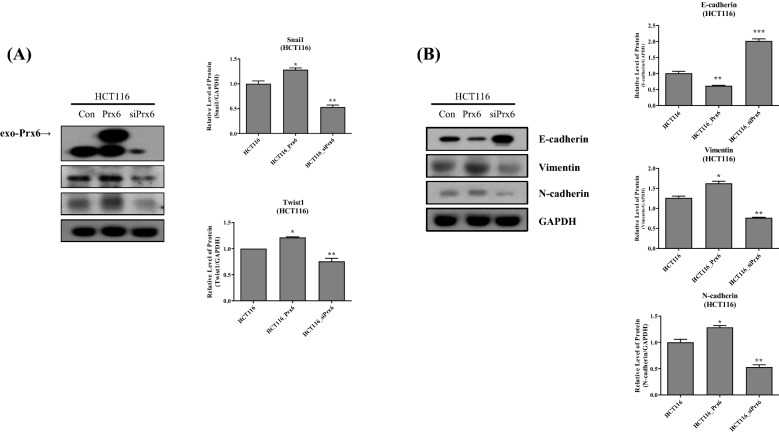
Prx6 regulates the expression of EMT-related genes in HCT116. **A** Prx6, Snail, and Twist1 expressions in HCT116 cells under Prx6 overexpressed or downregulated conditions by Western blotting. **B** Western blotting for EMT-marker proteins was conducted in HCT116 cells expressing either Prx6 or siPrx6 expressing cells. Graphs represent the quantification of Western blot band intensity. Data are expressed as mean ± SD (n = 3). *p < 0.05, **p < 0.01, and ***p < 0.001

We constructed Prx6-overexpressed HCT116 (HCT116_Prx6) by transfecting pLenti 6.3-Prx6 to confirm the Prx6 effect on the EMT signaling factors. Furthermore, the RNA silencing technique was used to downregulate Prx6 expression in HCT116 (HCT116_siPrx6). The expression of EMT-transcription factors, Snail and Twist1, increased under Prx6-overexpressed condition (Fig. 2A). Furthermore, the Prx6 downregulation showed decreased EMT-transcription factors in HCT116. In Prx6-overexpressed HCT116 cells, the epithelial marker protein, E-cadherin, decreased, whereas the mesenchymal marker proteins, Vimentin and N-cadherin, increased (Fig. 2B). Prx6 downregulation increased E-cadherin and decreased Vimentin and N-cadherin compared with the HCT116-negative control cells. Additionally, another well-known colon cancer cell line, SW480, was used to detect the regulation of EMT-related genes by Prx6 (Additional file 1: Figure S1). These data also supported that Prx6 modulates EMT-related genes in colon cancer cells. Consequently, Prx6 regulates the EMT signaling pathway by modulating EMT-related transcriptional repressors and mesenchymal genes in HCT116 colon cancer cells.

### Peroxiredoxin 6 regulates proliferation and tumor growth in HCT116

**Fig. 3:**
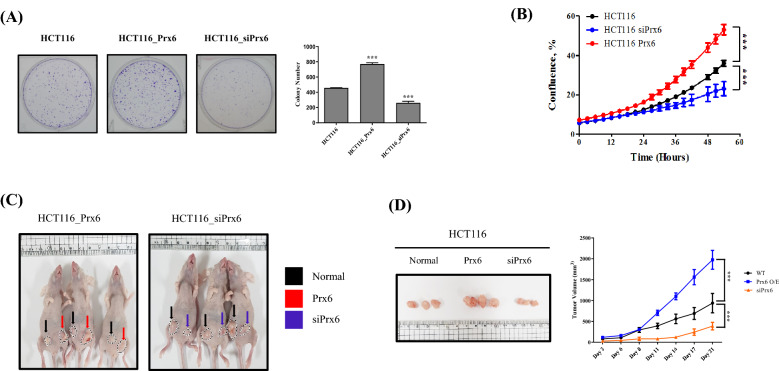
Prx6 regulates proliferation and tumor growth in HCT116. **A** Ten days after seeding HCT116 control, Prx6 overexpressing, and siPrx6 cells, they were stained with 5% crystal violet. The number of colonies from three independent experiments. **B** The proliferation rate of HCT116 Prx6 overexpressing cells or Prx6-knockdown cells were detected with IncuCyte until 60 h. **C** Photos of mice on day 21. Control, Prx6-overexpressed, siPrx6 HCT116 cells were intraperitoneally injected into nude mice. **D** Tumor volume was measured using a caliper at the appointed time. Data are expressed as mean ± SD (n = 3). *p < 0.05, **p < 0.01, and ***p < 0.001

We conducted a foci-formation assay to compare cancer colonization by Prx6 expression in colon cancer cells. Compared with normal cells, the number of colonies increased under Prx6-upregulated condition (Fig. 3A). In contrast with the Prx6 overexpression, silenced Prx6 expression decreased colony number. Furthermore, the proliferation assay was conducted to detect the Prx6 effect on colon cancer cells metastasis. Under the Prx6-overexpressed condition, HCT116 cells proliferation increased significantly (Fig. 3B).

Moreover, proliferation was decreased in siPrx6-treated cells. A xenograft assay also supported that Prx6 regulates colon cancer growth. The HCT116 control, HCT116_Prx6, and HCT116_siPrx6 cancer cell lines were subcutaneously injected into nude mice (Fig. 3C). Eleven days after HCT116 cell injection, Prx6 was overexpressed in the HCT116-injected mice, and the tumor volume increased significantly compared with that in the control mice (Fig. 3D). Therefore, the regulation of Prx6 expression affected the growth and proliferation of colon cancer cells.

### Peroxiredoxin 6 regulates EMT-related target genes through p38 phosphorylation

**Fig. 4:**
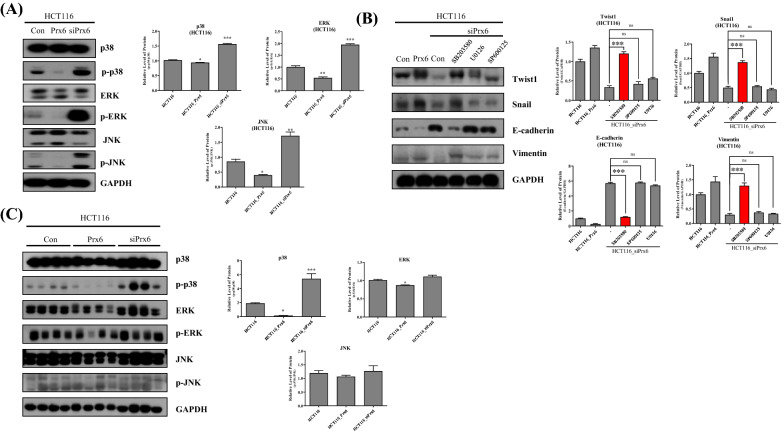
Prx6 regulates p38-mediated EMT-related genes. **A** Western blotting of MAPKs—p38, ERK, and JNK—in HCT116 cells in the presence or absence of Prx6. **B** The MAPK inhibitors were used in Prx6 downregulated HCT116 cells to detect the rescue of EMT-transcription factors and protein markers. Western blotting was used for detecting relative protein expressions. **C** The MAPK protein level was detected from tumor tissues via Western blotting. Graphs represent the quantification of Western blot band intensity. Data are expressed as mean ± SD (n = 3). *p < 0.05, **p < 0.01, and ***p < 0.001

Studies have demonstrated that p38 inhibits EMT by maintaining E-cadherin through TAK1 and NF-Kb modulation [25]. Therefore, we confirmed whether Prx6 regulates EMT signaling through p38 regulation. First, the MAPKs expression level was detected in Prx6 overexpressed and silenced HCT116 cells (Fig. 4A). The phosphorylation of p38, ERK, and JNK decreased under Prx6-upregulated conditions. In contrast with Prx6 overexpression, the downregulation of Prx6 increased p38, EKR, and JNK phosphorylation. SW480 cells also show a similar tendency in MAPK expression under Prx6 overexpressed and silenced conditions (Additional file 1: Figure S2A). We used representative MAPK inhibitors SB203580 (a p38 inhibitor), SP600125 (a JNK inhibitor), and U0126 (an ERK inhibitor) to confirm which MAPKs affected the EMT-related genes. Each MAPKs inhibitor was treated in Prx6-silenced HCT116 cells, and Western blotting was performed to the expression of EMT-related genes (Fig. 4B). Interestingly, the downregulated mesenchymal gene expression under the siPrx6 condition increased with the p38 inhibitor SB203580 treatment. Furthermore, E-cadherin expression, a representative epithelial gene, decreased under the p38-inhibited condition. Inhibition of p38 under siPrx6 condition also elevated mesenchymal genes and declined epithelial genes in SW480 cells (Additional file 1: Figure S2B). Next, we confirmed the MAPK expression level in tumor tissue harvested from the xenografted mice. Unlike ERK and JNK, p38 phosphorylation was significantly regulated by Prx6 expression (Fig. 4C). Hence, Prx6 regulates EMT signaling through p38 phosphorylation in colon cancer cells.

## Discussion

This study found that Prx6 promotes EMT in colon cancer, causing its growth and proliferation. It is the only 1-Cys peroxiredoxin with a glutathione peroxidase function. It has been shown that increased Prx6 expression helps cancer grow and proliferate. However, its specific role in colon cancer is still unknown. As an antioxidant enzyme that scavenges hydroperoxide, Prx6 protects cancer cells from the damage and cell death caused by oxidative stress [26]. In contrast, the reduction of Prx6 expression increases cell death due to oxidative stress [27]. Therefore, the proper regulation of the Prx6 level can be a critical factor in anticancer research. EMT is an essential process that helps developing cells have a more invasive phenotype during cancer progression [28]. Furthermore, ΕΜΤ makes cancer cells migratory, invasive, and resistant to chemotherapy [29]. For that reason, EMT is considered to play a significant role in cancer development and metastasis. During EMT, epithelial cells undergo molecular changes, including EMT-inducing transcription factors, such as Snai1, Twist1, and Zeb1. Also, the expression of epithelial marker proteins, such as E-cadherin, a component of the cell junction, is reduced, whereas the expression of mesenchymal marker proteins, such as Vimentin, increases [30]. Therefore, EMT signaling is one of the most critical factors of cancer progression and metastasis. Additionally, graft Prx6 onto EMT signaling is probably a new cancer therapy target.

In this study, the HCT116 and SW480 cells with high expression of Prx6 showed the highest occurrence of EMT compared with other colon cancer cells. Therefore, we obtained an apparent effect by changing the Prx6 expression in HCT116 (Fig. 1). Through Prx6 overexpression and knockdown in HCT116 and SW480 cells, the effect of Prx6 on EMT was elucidated in detail. Also, Prx6 overexpression in HCT116 and SW480 cells increased the EMT-transcription factors, promoting EMT. In contrast, Prx6 knockdown in HCT116 and SW480 cells inhibited EMT by inhibiting EMT-transcription factors (Fig. 2 and Additional file 1: Figure S1). Furthermore, we detected proliferation and tumor growth in Prx6-upregulated or -downregulated HCT116 cells. From Figs. 1 and 2, Prx6 and EMT-related gene expressions were more attracted in HCT116 than SW480. Therefore, we used HCT116 cells to detect proliferation and tumor growth of Prx6. It was confirmed that foci formation and proliferation of cells were more increased and EMT promotion in Prx6-overexpressed HCT116. In contrast, in HCT116 cells, where Prx6 was knocked down, foci formation and proliferation of cells decreased along with EMT inhibition (Fig. 3). Furthermore, in the xenograft experiments with nude mice, Prx6 overexpressed in the HCT116 cells showed more significant and faster growth than control HCT116 cells, whereas the Prx6-knockdown HCT116 had the opposite pattern.

The MAPKs can be associated with various cellular cycles, including growth, proliferation, and survival [22]. Representative MAPKs, p38, ERK, and JNK, which play different roles in cancers, are related to several mechanisms that regulate cancer progression [31]. Hydroperoxides activate and induce MAPKs through several signaling pathways. However, excessive hydroperoxides generation causes unnecessary phenomena, such as cancer growth. Here, Prx6 is one of the most effective antioxidants with anticancer abilities through hydroperoxide removal. Therefore, we examined the expression of MAPK dependent on Prx6 regulation in HCT116 and SW480 cells. Interestingly, all MAPKs were reduced in Prx6-overexpressed HCT116 and SW480 cells, whereas the silencing of Prx6 resulted in a significant MAPK increase in HCT116 and SW480 cells (Fig. 4 and Additional file 1: Figure S2). Furthermore, to determine which MAPKs are involved in EMT regulation by Prx6, the HCT116_siPrx6 and SW480_siPrx6 cells were treated with p38 phosphorylation inhibitor SB203580, ERK phosphorylation inhibitor U0126, and JNK phosphorylation inhibitor SP600125. The treatment with the p38 inhibitor SB203580 in HCT116_siPrx6 and SW480_siPrx6 cells dramatically increased EMT-transcription factors, attaining similar levels of HCT116_Prx6 and SW480_siPrx6. Additionally, changes in EMT-marker proteins were like those of HCT116_Prx6 and SW480_siPrx6. Thus, MAPK expressions in tumor lysate harvested from nude mice xenografts suggest that Prx6 regulates EMT through p38 phosphorylation.

Therefore, further studies on the concrete signaling pathway among Prx6, EMT, and p38 are warranted. The roles, pathways, and factors by which Prx6 directly regulates EMT signaling are critical research avenues for future studies. Moreover, we demonstrated that Prx6 regulation affects cancer progression through EMT signaling modulation, we believe that the effect of Prx6 regulation on cancer metastasis also needs to be further studied.

## Conclusions

Prx6 plays a critical role in proliferation and tumorigenesis in colon cancer cells. We demonstrated that Prx6 regulated colon cancer cell growth through modulating the EMT signaling pathway. Furthermore, we found that regulating EMT-related genes by Prx6 is mediated by p38 phosphorylation. Studies have suggested that regulating the EMT signaling pathway plays an important role in preventing cancer metastasis. Therefore, proper regulation of EMT is a key method for anticancer therapy. Thus, Prx6 plays an important role in anticancer treatments due to EMT regulation.

## Supplementary Information

**Additional file 1: Figure S1**. Prx6 regulates the expression of EMT-related genes in SW480. Prx6, Snail, and Twist1 expressions in SW480 cells under Prx6-overexpressed or downregulated conditions using Western blotting. Western blotting for EMT-marker proteins was conducted in SW480 cells expressing either Prx6 or siPrx6 expressing cells. Graphs represent the quantification of Western blot band intensity. Data are expressed as mean ± SD (n = 3). *p < 0.05, **p < 0.01, and ***p < 0.001. **Figure S2**. Prx6 regulates the p38-mediated EMT-related genes in SW480. (A) Western blotting of MAPKs—p38, ERK, and JNK—in SW480 cells in the presence or absence of Prx6. (B) The MAPKs inhibitors were used in Prx6 downregulated SW480 cells to detect the rescue of EMT-transcription factors and protein markers. Western blotting was used to detect relative protein expressions. Graphs represent the quantification of Western blot band intensity. Data are expressed as mean ± SD (n = 3). *p < 0.05, **p < 0.01, and ***p < 0.001.

## Data Availability

All data generated and analyzed during this study are presented in the manuscript. Please contact the corresponding author for access to the data presented in this study.

## Abbreviations

Prx6: Peroxiredoxin 6
EMT: Epithelial–mesenchymal transition
ROS: Reactive oxygen species
MAPKs: Mitogen-activated protein kinases
