# Supplementary material for: Peroxiredoxin-6 regulates p38-mediated epithelial–mesenchymal transition in HCT116 colon cancer cells
Source: J Biol Res (Thessalon). 2021 Nov 23;28:22. doi: 10.1186/s40709-021-00153-6 (PMC8609821; doi:10.1186/s40709-021-00153-6)
Supplement: Supplementary file 1 — Additional file 1: Figure S1. Prx6 regulates the expression of EMT-related genes in SW480. Prx6, Snail, and Twist1 expressions in SW480 cells under Prx6-overexpressed or downregulated conditions using Western blotting. Western blotting for EMT-marker proteins was conducted in SW480 cells expressing either Prx6 or siPrx6 expressing cells. Graphs represent the quantification of Western blot band intensity. Data are expressed as mean ± SD (n = 3). *p < 0.05, **p < 0.01, and ***p < 0.001. Figure S2. Prx6 regulates the p38-mediated EMT-related genes in SW480. (A) Western blotting of MAPKs—p38, ERK, and JNK—in SW480 cells in the presence or absence of Prx6. (B) The MAPKs inhibitors were used in Prx6 downregulated SW480 cells to detect the rescue of EMT-transcription factors and protein markers. Western blotting was used to detect relative protein expressions. Graphs represent the quantification of Western blot band intensity. Data are expressed as mean ± SD (n = 3). *p < 0.05, **p < 0.01, and ***p < 0.001. [file 40709_2021_153_MOESM1_ESM.pptx]

## Slide 1
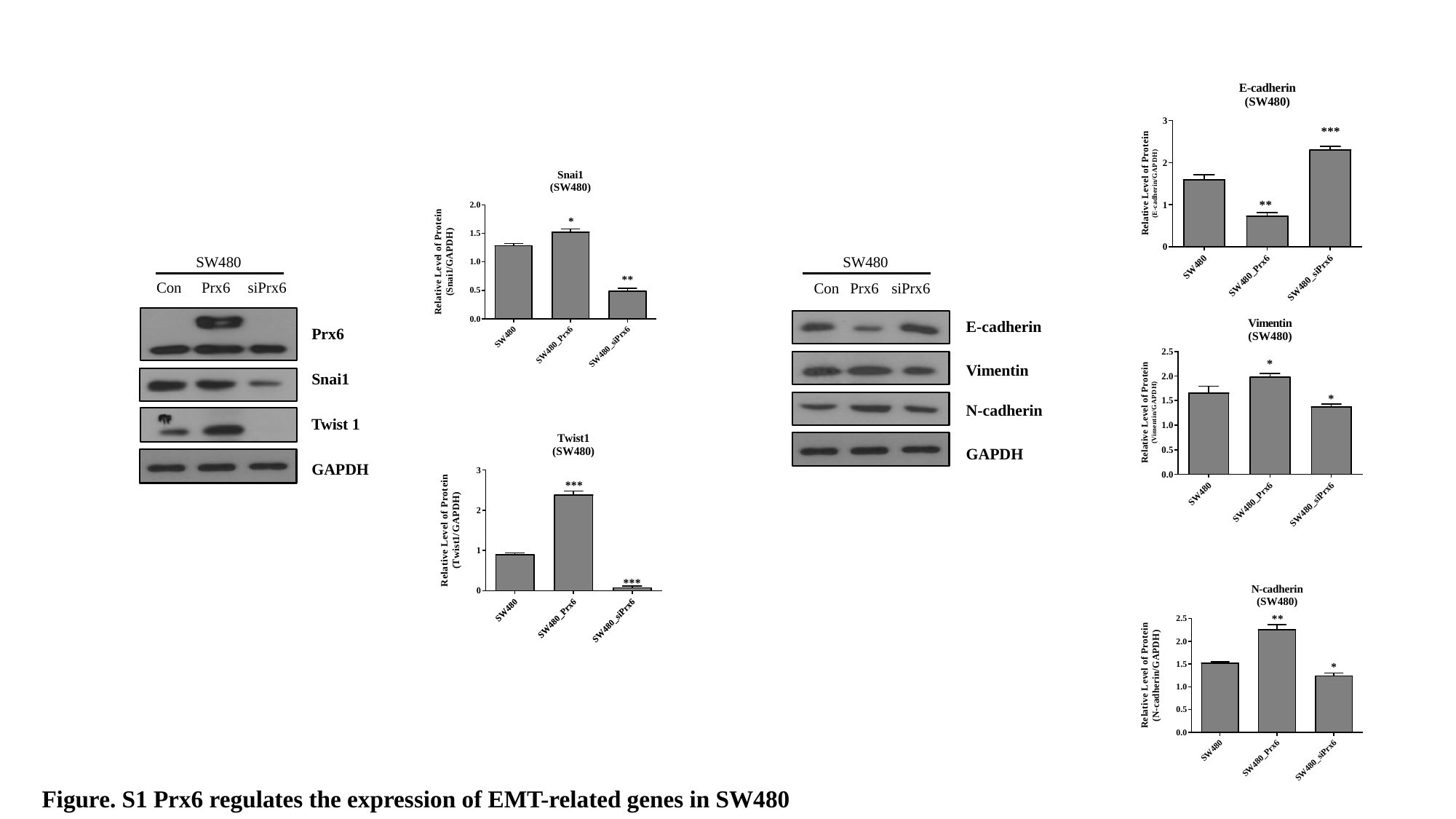

SW480
SW480
 Con
 Prx6
 siPrx6
 Con
 Prx6
 siPrx6
E-cadherin
Prx6
Vimentin
Snai1
N-cadherin
Twist 1
GAPDH
GAPDH
Figure. S1 Prx6 regulates the expression of EMT-related genes in SW480

## Slide 2
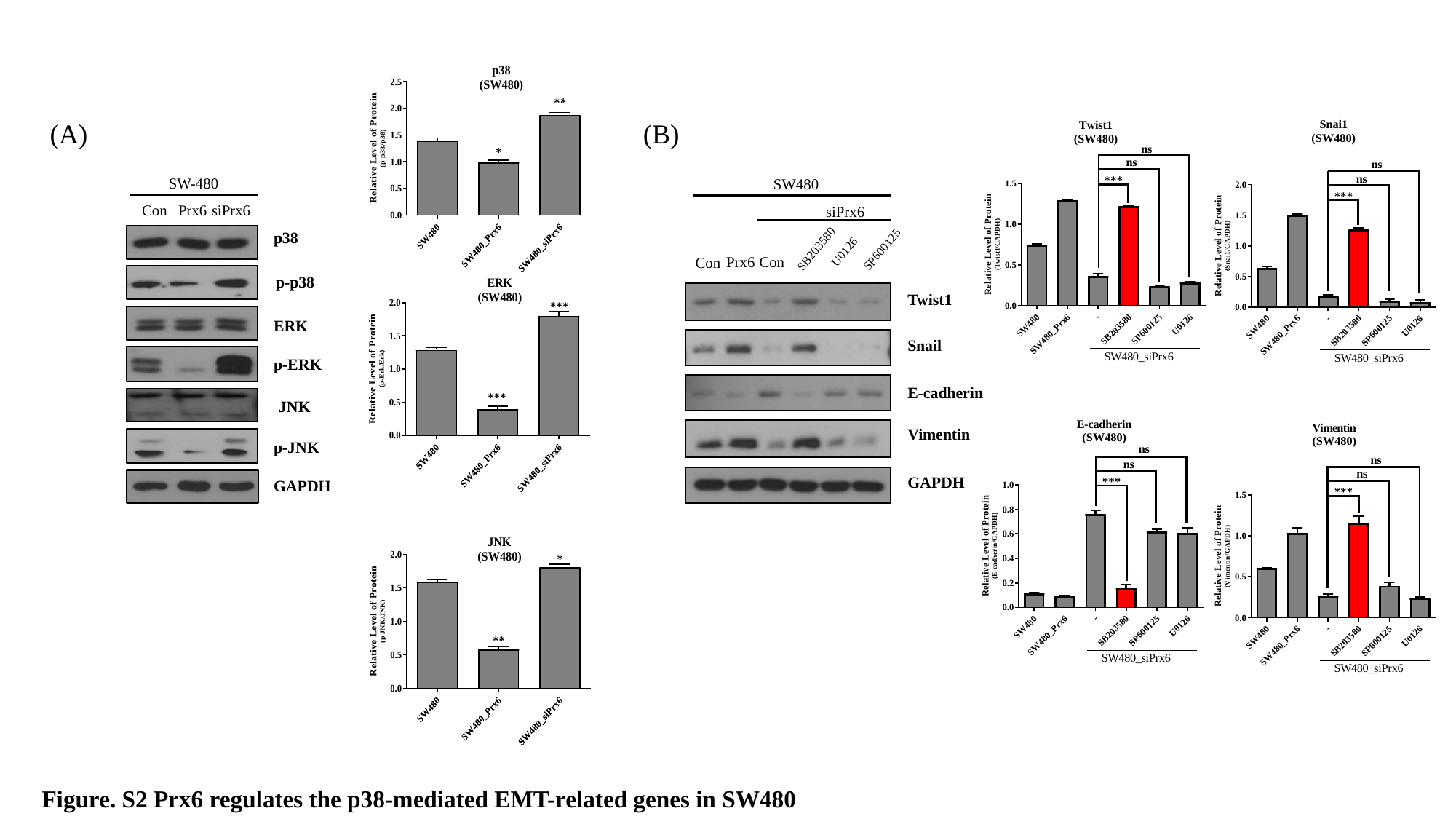

(A)
(B)
SW-480
SW480
 Con
 Prx6
 siPrx6
 siPrx6
p38
 SB203580
SP600125
 U0126
 Prx6
 Con
 Con
p-p38
Twist1
ERK
Snail
p-ERK
E-cadherin
JNK
Vimentin
p-JNK
GAPDH
GAPDH
Figure. S2 Prx6 regulates the p38-mediated EMT-related genes in SW480
